# Observations of lightning in relation to transitions in volcanic activity during the 3 June 2018 Fuego Eruption

**DOI:** 10.1038/s41598-020-74576-x

**Published:** 2020-10-22

**Authors:** Christopher J. Schultz, Virginia P. Andrews, Kimberly D. Genareau, Aaron R. Naeger

**Affiliations:** 1 NASA’s Short-Term Prediction and Research Transition Center, Marshall Space Flight Center, Huntsville, AL 35812 USA; 2 Department of Geological Sciences, The University of Alabama, Box 870338, Tuscaloosa, AL 35487 USA; 3 Earth System Science Center, The University of Alabama in Huntsville, Huntsville, AL USA

**Keywords:** Atmospheric dynamics, Natural hazards

## Abstract

Satellite and ground-based remote sensing are combined to characterize lightning occurrence during the 3 June 2018 Volcán de Fuego eruption in Guatemala. The combination of the space-based Geostationary Lightning Mapper (GLM) and ground-based Earth Networks Total Lightning Network observed two distinct periods of lightning during this eruption totaling 75 unique lightning flash occurrences over five hours (57 in cloud, 18 cloud-to-ground). The first period of lightning coincided with the rapid growth of the ash cloud, while the second maxima occurred near the time of a deadly pyroclastic density current (PDC) and thunderstorm. Ninety-one percent of the lightning during the event was observed by only one of the lightning sensors, thus showing the importance of combining lightning datasets across multiple frequencies to characterize electrical activity in volcanic eruptions. GLM flashes during the event had a median total optical energy and flash length of 16 fJ, and 12 km, respectively. These median GLM flash energies and lengths observed in the volcanic plume are on the lower end of the flash spectrum because flashes observed in surrounding thunderstorms on 3 June had larger median total optical energy values (130 fJ) and longer median flash lengths (20 km). All 18 cloud-to-ground flashes were negative polarity, supportive of net negative charge within the plume. Mechanisms for the generation of the secondary lightning maxima are discussed based on the presence and potential interaction between ash plume, thunderstorm, and PDC transport during this secondary period of observed lightning.

## Introduction

Lightning is one indicator of explosive volcanic eruptions^1–17^. There are two primary mechanisms hypothesized to be responsible for charge generation during volcanic events which may result in lightning. Electrification can occur because of fractoemission as magma is jettisoned from the vent and enters into the near-vent region of the ash plume^18^. The process of brittle fragmentation creates ionized pieces of the previously existing particles. Triboelectrification also occurs through collision and transfer of charge between ash particles in the rising plume^7,19–21^. Once ash has reached the mixed phase region, supercooled liquid water nucleates as ice on ash grains^8,17,22,23^. As the ash-laden ice grows in the mixed phase microphysical environment, rebounding collisions can occur between the hydrometeors, and charge may be transferred through additional processes like the non-inductive charging mechanism (NIC)^24–27^. Lightning discharges occur as the electric field reaches a critical breakdown magnitude, similar to how thunderstorms generate lightning^6,8,10,12^. The electrification mechanism and efficiency of charge separation within the rising ash plume will control the length of resulting lightning discharges, which tend to increase with greater altitude^10^.

In thunderstorms, sudden increases in lightning signal an increase in the size and magnitude of the thunderstorm updraft volume^28–32^. This sudden increase is well correlated to severe weather occurrence^33,34^. Furthermore, lightning flash size (area and/or length) is a diagnostic to identify regions of turbulence within a storm cloud. Smaller sized flashes occur in turbulent updraft regions, while larger flashes are observed in more laminar regions^30,35,36^. Flash size as an indicator of turbulence has been extended to volcanic plumes through the use of Lightning Mapping Arrays (LMAs) during the Redoubt (Alaska, U.S.A.) eruption in 2009^9,10,12^. The LMA observations indicated that the flash energy and area changed throughout the eruption due to variations in turbulence, with more turbulent portions of the eruption column corresponding to lower flash energy and area.

Therefore, the goal of this study is to characterize lightning observed in a volcanic plume during growth stages of the plume and near the time of a deadly pyroclastic density current (PDC) during the 3 June 2018 Fuego eruption. This is accomplished using a combination of space based Geostationary Lightning Mapper (GLM)^37,38^ data, ground based Earth Networks Total Lightning Network (ENTLN)^39^ data, and visible imagery from the GOES-16 Advanced Baseline Imager (ABI)^40^. One primary objective is to examine the evolution of the lightning observed throughout the 3 June 2018 eruption to determine how the lightning data aligns with key changes in volcanic activity (e.g., plume growth, PDC generation). Additionally this paper assesses GLM flash lengths and total optical energy of lightning events within the volcanic plume and compares with flash length and flash energy observed in thunderstorms. A final objective is to demonstrate how GLM and ENTLN provide unique information on lightning during an explosive eruption, that when combined, provide a more complete picture on the evolution of lightning during these hazardous volcanic events.

## 2018 Eruption of Volcán de Fuego, Guatemala

Volcán de Fuego is an active stratovolcano, located in central Guatemala as part of the Fuego-Acatenango volcanic complex^41–43^. The Fuego eruption on 3 June 2018 (Table 1) was a relatively short duration explosive eruption that displaced more than 12,000 people and resulted in 165 confirmed deaths and 260 missing, based on reports of the National Coordination for Disaster Reduction of Guatemala (CONRED), Instituto Nacional de Sismología, Vulcanologia, Meteorologia e Hidrología (INSIVUMEH) and the American Red Cross. According to the Global Volcanism Program, the eruption had a volcanic explosivity index (VEI) of 3 out of 8 and the ash plume reached ~ 10 km above the vent. A large portion of the devastation from this eruption was caused by pyroclastic density currents (PDCs), which are a high-temperature mix of gases, ash, lapilli, and bombs. The Fuego PDCs traveled 10 km from the summit at 700 °C with secondary ash plumes reaching 6 km above the ground surface. Volcanic lightning can occur in PDCs^44^, but has not been previously observed by satellites. Increases in lightning due to propagation of PDCs during the 2015 Calbuco eruption in Chile were suggested in an analysis^16^ using World Wide Lightning Location Network (WWLLN)^45^ data.

**Table 1:** 3–4 June 2018 Volcán de Fuego eruption timeline from reports of the Volcanic Ash Advisory Center (VAAC), National Coordination for Disaster Reduction of Guatemala (CONRED), and Instituto Nacional de Sismología, Vulcanologia, Meteorologia e Hidrología (INSIVUMEH).

3–4 June 2018 Volcán de Fuego eruption timeline	
Time UTC	Event description
**3 June 2018**	
14:00	First eyewitness reports of eruption
16:42	Volcanic ash emitted but not observed by GOES-East
16:51	Volcanic ash emitted but not observed by GOES-East
18:14	First volcanic lightning observed by GLM
18:15	Beginning of explosive volcanic eruption observed by GOES-East
18:32	First lightning observed by ENTLN
19:40	Continuous eruption with associated small PDC
21:43	Last volcanic lightning recorded by ENTLN
21:45	Continuous eruption with associated PDC
22:00	Major PDC reported, which traveled 8–10 km at 700 °C with secondary ash plumes reaching 6 km above the surface
22:05	Last volcanic lightning recorded by GLM
22:12	Continuous eruption with ash reported in wells to the east of the summit and possible secondary explosion
**4 June 2018**	
03:45	Final PDC or first lahar reported
04:10	Volcanic ash emissions dissipated

*GOES* Geostationary Observing Environment Series, *GLM* Geostationary Lightning Mapper, *ENTLN* Earth Networks Total Lightning Network, *PDC* pyroclastic density current.

## Lightning detection

The launch of GOES-16 and GOES-17 GLMs on the GOES-R series of satellites could potentially improve the scientific understanding of lightning in volcanic columns and plumes due to its consistent location in orbit above North, Central, and South America, allowing for the development of more advanced volcano warning systems. The GLM has an 8 km × 8 km spatial resolution at nadir, a 2 ms temporal resolution, and detects lightning in a 1 nm window centered on 777.4 nm^37,38^. The field of view of GLM is ± 54° latitude and GOES-16 is located at longitude of 75.2° W. GLM level-2 data files are generated every 20 s, containing information about flash, group, and event location, along with flash characteristics like flash area and flash total optical energy.

ENTLN flash data are used to illustrate lightning activity from the perspective of a ground based lightning location system during the 3 June 2018 Fuego eruption. ENTLN operates in the electromagnetic spectrum between 5 kHz and 10 MHz and differentiates between intra-cloud (IC) and cloud-to-ground lightning (CG)^39^. When compared with other global lightning location systems, ENTLN has the best-reported detection efficiency in the area of the Fuego volcano at the time of the eruption^46^.

## Results

**Figure 1:**
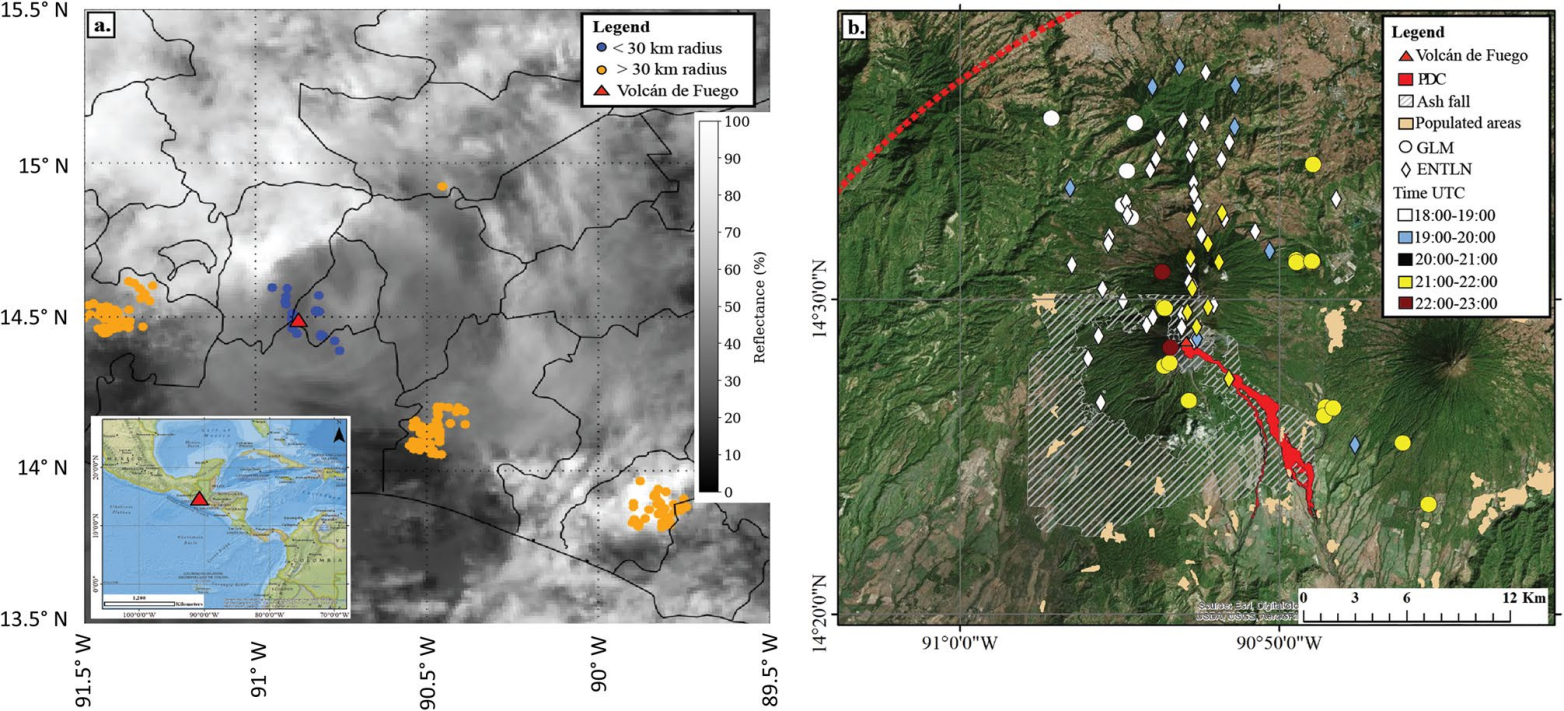
(**a**) GOES channel 1 (0.47 µm) reflectance with GLM lightning data overlaid (blue < 30 km from vent, orange > 30 km from vent). Lower reflectance in the area of the blue dots is associated with the ash plume while higher reflectance in areas with orange dots is associated with ‘ash-free’ convective clouds. Insert shows the location of Volcán de Fuego marked with the red triangle. (**b**) GLM (circles) and ENTLN (diamonds) data by time displayed over Esri world imagery during the 3 June 2018 Volcán de Fuego eruption. The dashed red line is an approximation of the ash plume using MODIS true color imagery at ~ 19:00 UTC. A Copernicus EMS map and vector data were used to obtain PDC extent, ash fall, and the location of populated areas.

**Figure 2:**
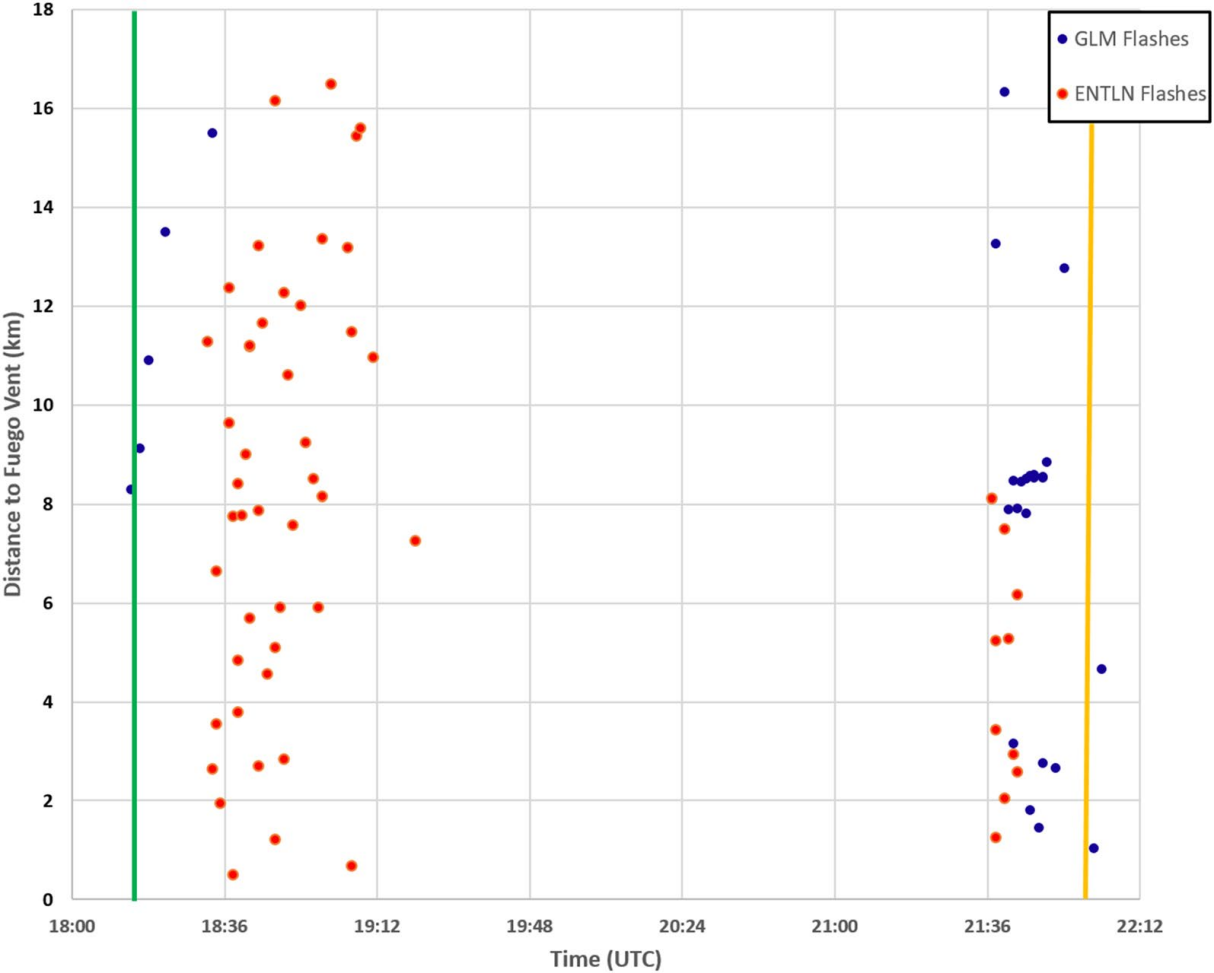
GLM (blue) and ENTLN (red) detected lightning occurrences through time within a 30 km radius of the Fuego vent. The vertical, green line on the left represents the approximate start of the explosive eruption based on the appearance of the overshooting top in the 0.47 µm satellite imagery from GOES-16 at 18:15 UTC. The orange vertical line is the report time of 22:00 UTC for the PDC associated with Fuego on 3 June 2018.

GLM flash locations between 18:00 and 23:00 UTC on 3 June 2018 are displayed over GOES ABI Channel 1 0.47 µm visible imagery (Fig. 1a) to show the lightning flashes observed by the GLM in Mexico and Central America. Channel 1 was chosen because it’s the primary visible band for monitoring aerosols like volcanic ash, and is useful in distinguishing aerosols from water and ice clouds. Four distinct clusters of lightning were observed in the region during this five-hour period, with 75 lightning flashes observed by GLM and ENTLN within 30 km of Fuego. Figure 1b shows of lightning from GLM (circles) and ENTLN (diamonds) data displayed over Esri ArcGIS world imagery by time within 20 km of the volcano. The lightning from GLM and ENTLN during the eruption occurred two distinct periods: 18:14–19:21 UTC and 21:37–22:05 UTC (Fig. 2). GLM observed six flashes during the first electrically active period, and 22 flashes during the second electrically active period (Tables S1 and S2). ENTLN observed 44 lightning flashes during the first period and 10 lightning flashes during the second period. Interestingly, 76% of these flashes remained in the cloud.

Only seven of the 75 total flashes observed by the two systems were observed GLM and ENTLN at the same time. ENTLN had 47 flashes that GLM did not observe, while GLM had 21 flashes that ENTLN did not observe. This means 91% of the lightning during the eruption was only seen by one of the two lightning sensors at any given time. GLM measurements indicated a different time for the onset and cessation of lightning in the volcanic column/plume compared to ENTLN, while ENTLN observed lightning within the plume that GLM did not detect. Therefore, the combination of the ground-based and satellite-based lightning observations provided a more complete picture of the electrical evolution of the Fuego eruption.

### Lightning during the explosive plume development stage

**Figure 3:**
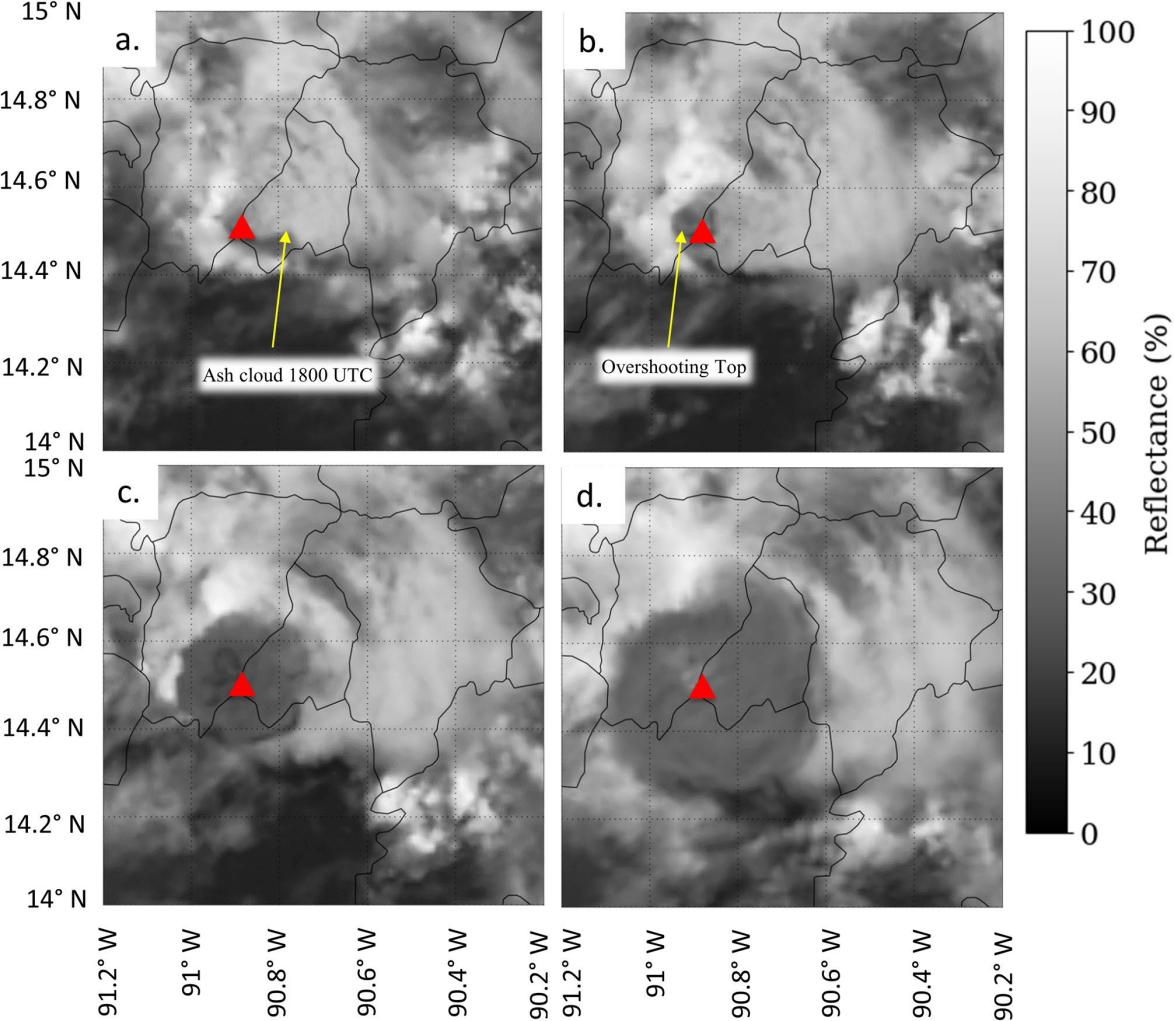
GOES channel 1 (0.47 µm) reflectance imagery on 3 June 2018 at approximately (**a**) 18:00 UTC, (**b**) 18:15 UTC, (**c**) 18:30 UTC, and (**d**) 18:45 UTC. The red triangle indicates the location of the Fuego volcano.

**Figure 4:**
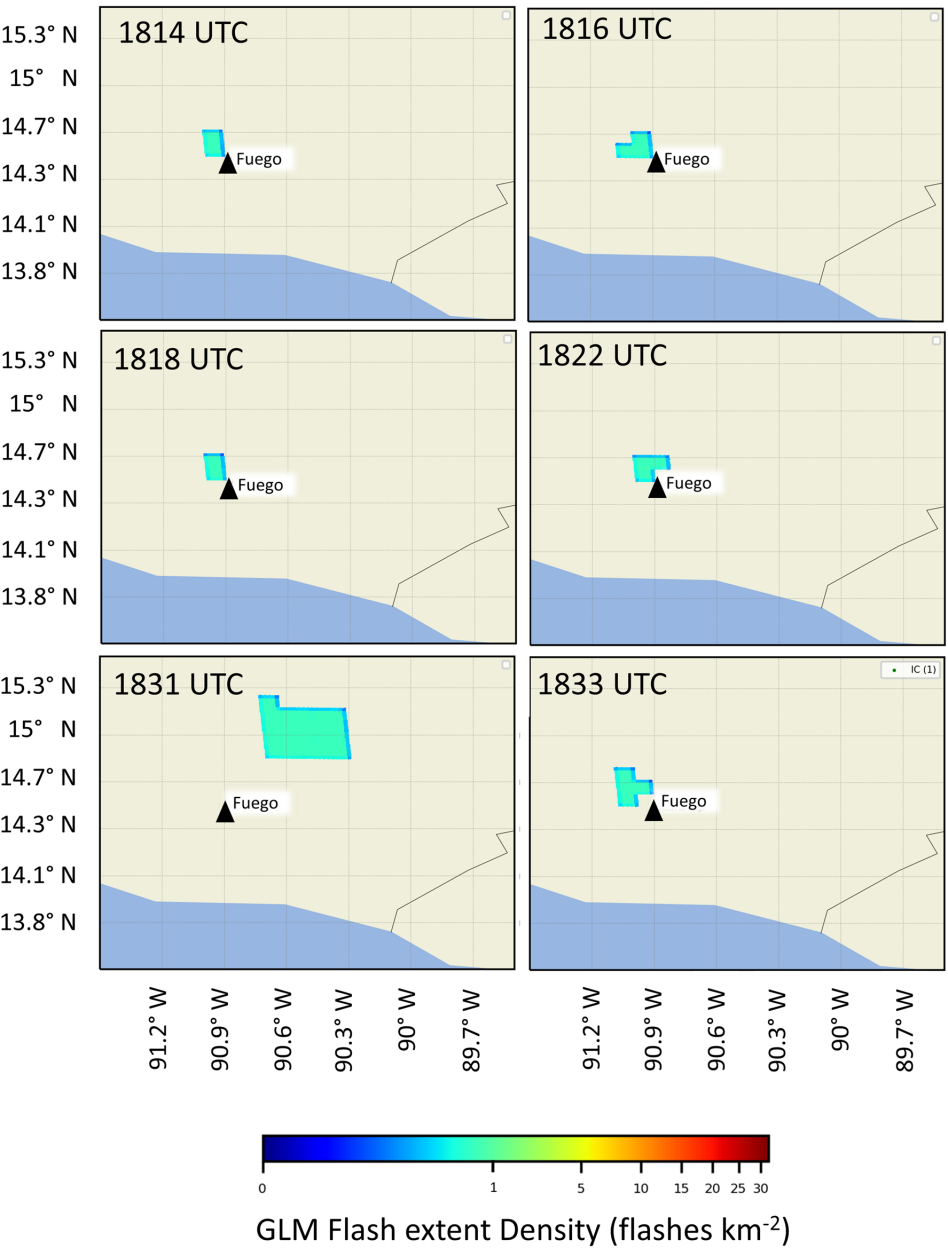
Flash extent density plots of GLM data from between 18:14 UTC and 18:33 UTC using GLMTools software^62^. The black triangle indicates the location of the Fuego Volcano.

The earliest detections of lightning in the plume were made by GLM at 18:14 UTC as the ash cloud took on the appearance of an overshooting top^47,48^ at 18:15 UTC (Figs. 2 and 3a,b). Overshooting tops are domelike protrusions associated with strong vertical motion that penetrates above the equilibrium level at the interface of the troposphere and stratosphere. GLM observed additional flashes through 18:33 UTC. Five of the six flashes observed during this period by GLM occur in the region where the overshooting top of the plume was observed in the 0.47 µm channel of GOES-16′s ABI (Fig. 4). The remaining flash was approximately centered 40 km to the northeast of Fuego in the lower ash cloud at 14.92 N, -90.46 W (Fig. 3c). This was the largest flash observed by GLM during the entire event, with a flash length of ~ 38 km and the brightest total optical energy of 1,857 fJ. Its position further away from the OT feature as compared to the other smaller flashes in the vicinity of the overshooting top matches theories on the relationship between flash size and kinematic processes^12,35,36^, where the largest flashes occur away from the most turbulent regions of a cloud. The mean GLM flash length was 14 km and the average total optical energy was 20 fJ for this growth stage.

The ENTLN observed lightning within the plume starting at 18:32 UTC, and detections of IC and CG discharges continued through 19:21 UTC (Fig. 2). ENTLN observed a total of 34 IC discharges and 10 CG discharges during this time. All 10 CG discharges were of negative polarity, with a mean peak current of -13.7 kA and maximum negative peak current magnitude of -26.8 kA. Only one single ENTLN discharge corresponded with a GLM flash at 18:33 UTC. This flash was identified as an IC discharge by ENTLN, and GLM indicates the flash had a length of 16 km, and a total optical energy value of 18 fJ.

The lack of lightning detection by GLM beyond 18:33 UTC was likely due to the optically thick ash cloud expansion by 18:30 UTC (Fig. 3c,d), which extinguished the optical signal from the lightning before it reached the GLM instrument. Both GLM and ENTLN detections were all to the north and northeast of the vent, and within the ash plume during this period (Fig. 1b).

### Lightning during the thunderstorm and PDC transport stage

**Figure 5:**
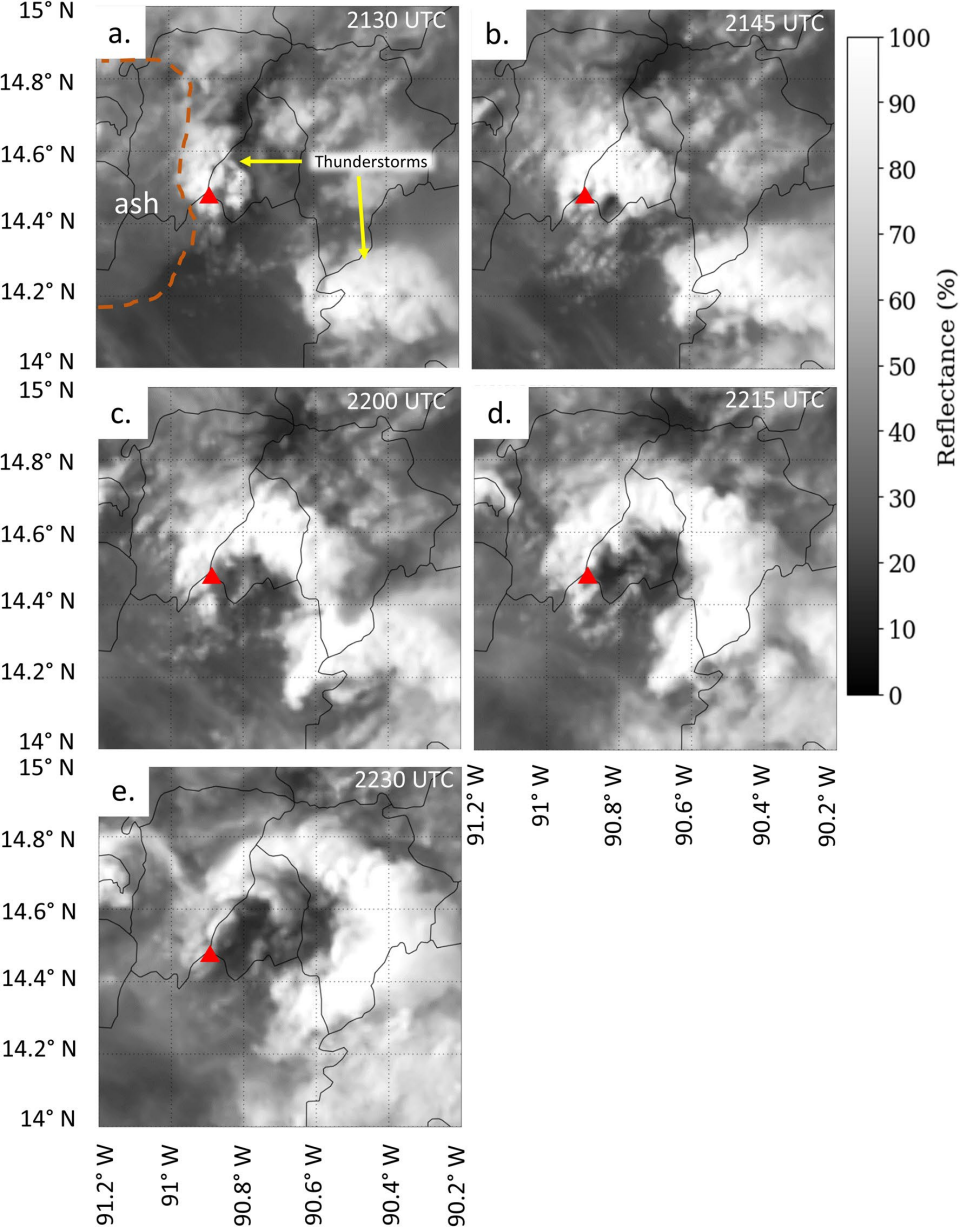
GOES channel 1 (0.47 µm) reflectance imagery on 3 June 2018 at approximately (**a**) 21:30 UTC, (**b**) 21:45 UTC, (**c**) 22:00 UTC, (**d**) 22:15 UTC and (**e**) 22:30 UTC. The orange dashed line indicates the lower reflectance due to ash near Fuego (red triangle).

At 21:37 UTC, a second period of lightning activity was observed near the volcano by both ENTLN and GLM (Fig. 2), with a total of 10 flashes and 22 flashes, respectively. Satellite imagery indicates that a small thunderstorm develops over Fuego around 21:30 UTC to 22:30 UTC (Fig. 5). This period is also near the time of the reported PDC transport at 22:00 UTC.

ENTLN observed 10 flashes between 21:37 UTC and 21:43 UTC, with a median distance of 4.3 km from the vent. Importantly, eight of the ten ENTLN lightning flashes during this period were negative CG flashes, with a mean peak current of -27.1 kA and a maximum peak current magnitude of -42.6 kA. Twenty-two flashes were observed by GLM between 21:38 and 22:03 UTC. GLM flash length was less than 21 km with an average of 13 km and flash energies were less than 120 fJ with an average of 28 fJ. All 22 flashes were within 16 km of the vent, with a median distance of 8.4 km.

Sixteen of the GLM flashes were to the north of the vent, while the remaining six of the flashes were along the southern and eastern flanks in the direction of PDC transport (Fig. 1b). The first two of these six GLM flashes during the period are 13 and 16 km away from the vent at 21:38 and 21:40 UTC. The next four flashes in identified to the east-southeast of the volcano gradually increased in distance. The first flash at 21:42 UTC was 3 km from the vent and the last flash to east-southeast of the volcano was 9 km at 21:50 UTC (Table S2). Unfortunately, it is not possible to discern the exact generation source of these lightning flashes because of the co-location of the thunderstorm, the volcanic plume, and the PDC transport. The temporal resolution of the satellite information is too temporally coarse to resolve cloud feature development, there is limited reporting of the PDC event during this second period of lightning, and height information does not exist for the lightning data^11^.

### Comparison of flash characteristics with tropical thunderstorms on 3 June 2018

**Figure 6:**
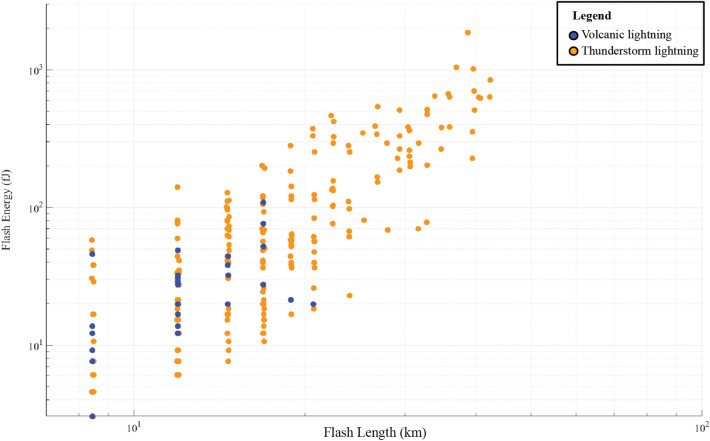
A comparison of GLM-determined flash length (km) versus flash energy (fJ) during the 3 June 2018 Fuego eruption, where the blue symbols indicate volcanic lightning and the orange symbols indicate lightning within 120 km of Fuego.

Next is a need to quantify the flash area and total optical energy to understand the lightning associated with the eruption fits within the spectrum of flashes observed by GLM in thunderstorms. Figure 6 illustrates the relationship between flash length and flash energy for lightning within a ~ 120 km radius of the volcano between 18:00 and 23:00 UTC. Blue dots indicate lightning within a 30 km radius, which may be associated with the volcanic eruption, while orange dots are other lightning flashes from tropical thunderstorms located > 30 km from the volcano (Figs. 1a and 6). Flash lengths in the volcanic plume had a median of 12 km (n = 27). Total optical energy values had a median of 16 fJ, with a maximum of 1,857 fJ at 18:33 UTC. Every presumed volcanic lightning flash except the flash at 18:33 UTC occurred within 16 km from the vent, with a median distance of 8.5 km. For the thunderstorms occurring in the region on the afternoon of 3 June 2018, flash lengths were < 40 km with an average of 20 km and flash energies were < 1050 fJ with an average of 130 fJ (n = 239). The median flash area and total optical energy observed over land within the GOES-16 GLM field of view for a nine-month period in 2018 were 21 km and 230 fJ, respectively^37^. Thus, volcanic lightning observed in the Fuego eruption was optically weaker and smaller in size than both thunderstorm lightning in Guatemala on the same day and also with lightning observed in the GOES-16 GLM total field of view for most of the year.

### Charge structure of the 3 June 2018 Fuego eruption

Polarity information derived from ENTLN also supports a hypothesis that the plume contained a net negative charge. This hypothesis is supported by the fact that all 18 CG flashes contained negative polarity and zero positive CG flashes were observed during either phase of active lightning in the plume. Multiple charge structures are possible in thunderstorms^49^ and volcanic plumes^11,50,51^; and there is evidence that this potential may exist in the IC cloud information from ENTLN. While IC flashes do not have a polarity, the + IC and –IC designations can provide some context for the vertical direction of the negative leader propagation. + IC flashes means the negative leaders propagated upward into a positive charge regions, while the –IC flashes indicate that negative leaders propagated downward into positive charge region^49^. Given that 23 of the 36 lightning flashes were designated as + IC, there is indications of a positive charge region above a negative charge region. However, there were 13 –IC flashes, which would indicate negative charge above positive charge. Thus, without full three-dimensional LMA data or electric field balloon soundings, this study cannot fully resolve the charge structure present in the 3 June 2018 eruption from ENTLN alone.

## Discussion

### Use of lightning datasets to fill gaps in satellite imagery

Current methods by operational volcano monitoring efforts^52^ utilize lightning measurements to fill gaps in satellite data during the analysis of active volcanoes. The Fuego eruption from 3 June 2018 provides another example of how the lightning data can fill these gaps in anticipating plume growth. The temporal resolution of the GOES-16 full disk advanced baseline imager on 3 June 2018 over Guatemala was 15 min, which is too coarse to track rapid changes in cloud top features^53,54^. During rapid growth stages of the volcanic plume between 18:00 and 18:45 UTC, GLM observes flashes collocated with this column at 18:14, 18:16, 18:18, 18:22, and 18:33 UTC as the column rises into the atmosphere (Figs. 3 and 4). ENTLN begins observing lightning near the overshooting top feature at 18:32 UTC, and continues to identify lightning events within 16 km of the vent location through 19:21 UTC during continued growth of the plume.

### Hypotheses for the secondary lightning maxima between 2130 and 2205 UTC

The observations from this event provide the opportunity to speculate on possible mechanisms driving the secondary period of lightning during the Fuego eruption. The most obvious explanation for the reactivation of lightning in the plume could be renewed ash emissions or an increase in mass eruption rate^15^. This idea is plausible given that nine of the ten ENTLN flashes and 16 of the 22 GLM flashes observed between 21:30 and 22:03 UTC were to the north of the vent, consistent with the lightning observed with the growth stage of the volcanic plume.

However, thunderstorm development over the volcano during the secondary maxima in lightning activity (Fig. 5) and PDC transport complicates the interpretation of the lightning data because all three features in previous events have been observed to generate lightning^15–17,44,55^. The additional six GLM flashes and one ENTLN flash were located east-southeast of the volcano, along the direction of PDC transport, and directly under the thunderstorm. There is not enough information in the lightning data to partition the lightning occurrences observed by GLM and ENTLN between the thunderstorm, reinvigoration of the ash emission, and PDC transport.

It is also worth noting how the thunderstorm cloud arced with time in Fig. 5 during this period. This arc in the clouds aligns with the direction and time of the reported PDC, where the cloud arc moves further to the southeast with time between 21:45 and 22:30 UTC. Note the dark spot immediately southeast of the volcano indicator in Fig. 5b, which is in agreement with the location of the PDC in Fig. 1B. This dark area expands with time through 2230 UTC. Video evidence from the 3 June 2018 eruption shows the PDC propagating down the side of Fuego and extending into the cloud base (https://www.youtube.com/watch?v=1BVl2GsrQ0g). The interaction between the PDC and the thunderstorm is difficult to characterize because of a lack of observations and is worth investigation in future studies of the 3 June 2018 event.

## Conclusions

This study examined the evolution of the 3 June 2018 Fuego eruption from the perspective of the GLM, ENTLN, and ABI instrumentation. Key observations were:

1. During the growth stage of the 3 June 2018 Fuego event, both GLM and ENTLN observed lightning within the growing ash plume. GLM observed five flashes that were collocated with an overshooting top feature on GOES-16′s 0.47 µm visible imagery: a feature indicative of vigorous upward motion. GLM ceased observing lightning events near Fuego as the plume became optically thick. However, ENTLN detections increased in frequency and continued to pinpoint the locations of lightning until 19:21 UTC when lightning activity ceased in the plume.
2. A secondary peak in lightning occurrence was observed between 21:30 UTC and 22:05 UTC. Twenty-two GLM flashes and 10 ENTLN flashes were observed during this period. Sixteen GLM flashes and nine ENTLN flashes were collocated with the ash plume during this secondary maxima in activity. The remaining six GLM flashes and one ENTLN flash were collocated with the direction of PDC transport. However, at the same time a small thunderstorm was identified using satellite data in this same area as PDC transport, thus its difficult to tell if these additional flashes were part of the ash cloud, the thunderstorm, or within the PDC itself.
3. Total optical energy is weaker and flash lengths are smaller for lightning in volcanic plumes versus lightning in thunderstorms on the same day in Central America. Median and maximum flash energy for the Volcán de Fuego eruption was 16 fJ and 1,857 fJ, respectively, while median and maximum flash lengths were 12 km and 38 km, respectively. These values were considerably smaller than the median flash lenghts of 20 km and total optical energy of 130 fJ observed in thunderstorms in the region on 3 June 2018.
4. All 18 CG lightning flashes observed by ENTLN during this event were of negative polarity, and 76% of the flashes observed were IC flashes.
5. GLM and ENTLN combined provide the most complete picture of electrical activity within the Fuego volcanic plume on 3 June 2018. 91% percent of the flashes within the eruption were only observed by one of the two lightning sensors. GLM measurements provide a different time for the onset and cessation of lightning in the volcanic column/plume compared to ENTLN. ENTLN provided continuous observations of lightning as the volcanic plume matured and the flashes were optically dim to the GLM sensor.

Additionally, hypotheses related to the sources of the secondary maxima in lightning near the time of PDC are discussed. However, without additional data, definitive conclusions could not be determined. Additional work is need to understand the dynamics of the event, potentially from a modeling perspective, to better understand the primary drivers of the secondary increase in lightning during the 3 June 2018 Fuego eruption and the physical interaction between the PDC and the thunderstorm.

## Data and methods

### Spaceborne lightning data

The GLM monitors total lightning during the day and night in the near infrared (777.4 nm band) part of the spectrum^38,39^. The GLM instrument is a 1372 × 1300 pixel charge coupled device that is in the geostationary orbit GOES-East Position of 75°W longitude. The nadir resolution of the instrument is 8 km by 8 km, with a resolution of ~ 9 km by 14 km at the edges of the field of view. GLM data consist of a three-tier hierarchy of events, groups, and flashes^38,39^. A GLM event is defined as the occurrence of a GLM single pixel exceeding the instrument background threshold during a 2 ms period. A GLM group is defined as the grouping of one or more simultaneous GLM events that occur in the same 2 ms period and are adjacent to each other. A GLM flash is defined as a set of GLM groups that are sequentially separated in time and space by no more than 330 ms and 16.5 km, respectively. The reported position of the GLM flash is the space-averaged location of all the GLM groups that make up the flash. Similarly, the GLM group location is the space-averaged location of all the GLM events that make up the GLM group. Data were obtained through the NOAA GOES-16 Validation Campaign web portal run by the Information Technology and Systems Center at the University of Alabama in Huntsville, and are available via Amazon Web Services: https://aws.amazon.com/blogs/publicsector/accessing-noaas-goes-r-series-satellite-weather-imagery-data-on-aws/.

### Ground-based lightning data

Lightning data from the ENTLN was utilized to quantify lightning activity as sensed by ground-based networks. The ENTLN operates in a frequency range of 5 kHz to 10 MHz and detects rapid changes in the vertical electric field to pinpoint the location of IC and CG flashes^27,39^. In an inter-comparison between all global ground-based lightning location sensors, ENTLN provided the optimal performance in Guatemala when compared to the Lightning Imaging Sensor aboard the Tropical Rainfall Measurement Mission (TRMM) satellite^46,56,57^. Furthermore, the ENTLN data stream presently includes the WWLLN^45,46,58,59^ to incorporate lightning data that is not readily observed in specific regions of the world due to the lack of lightning sensors^46^. WWLLN operates at frequencies between 3 and 30 kHz^45^. ENTLN data can be requested through Earth Networks directly and are not included in the supplemental materials because of their proprietary nature: https://www.earthnetworks.com/why-us/networks/lightning/.

Polarity information was also utilized from the ENTLN because it provides insight into the overall charge structure of the cloud. Importantly, + IC and –IC flash designations do not imply that the IC flashes contain a polarity because all IC flashes are net charge neutral^60^. The sign designation implies the direction of the flash propagation, where + IC is indicative of upward propagation, where –IC is indicative of downward flash propagation between regions of positive and negative charge within the electrified cloud^49,61^.

### Geostationary Operational Environmental Satellite (GOES-16) Advanced Baseline Imager (ABI)

ABI has a total of 16 different spectral bands and includes two visible channels and ten infrared channels^39^. The aerosol monitoring visible band, ABI channel 1 at 0.47 µm (Fig. 1a), was chosen to observe the difference in reflectance between the ash plume and ‘ash-free’ convective clouds located near the eruption, using Python 2.7. GOES-16 provides continuous geostationary monitoring and a time of 19:30 UTC was used in this study because it is near the time of the Moderate Resolution Imaging Spectroradiometer (MODIS)-Aqua pass, which was used to get an approximation of the ash plume extent outlined in Fig. 1b.

*Esri ArcGIS:* GLM and ENTLN data were loaded with Python into ArcGIS and were then displayed over Esri high-resolution world map imagery (Fig. 1b) sourced from: DigitalGlobe | Sources: Esri, DigitalGlobe, GeoEye, Earthstar Geographics, CNES/Airbus DS, USDA, USGS, AeroGRID, IGN, and the GIS User Community | Esri, HERE, Garmin. A Copernicus Emergency Management Service map and vector data were used to obtain PDC extent, ash fall, and populated areas and uploaded into ArcGIS. The location map (Fig. 1a insert) is an Esri National Geographic basemap. Figure 1a insert and 1b were created using ArcGIS® software by Esri. ArcGIS® and ArcMap™ are the intellectual property of Esri and are used herein under license. Copyright © Esri. All rights reserved. For more information about Esri® software, please visit www.esri.com.

### Data processing

MATLAB R2018b, in combination with Python (2.7 and 3.7) data processing, was used in order to obtain statistically significant relationships between flash length (the square root of flash area^34^), flash energy, flash rate, and time. Flash length was used instead of flash area in order to relate the data to previous studies (Fig. 4). MATLAB R2018b was also used to calculate the distance in degrees and the azimuth angles in degrees from Fuego using great circle arcs (Fig. 2 and Figs. S1 and S2, Table S1) and to display the data. Both ENTLN (n = 54) and GLM (n = 27) lightning data are plotted as a function of time and distance in degrees from the volcanic vent.

## Supplementary information

Supplementary Information.

## Data Availability

GLM and ABI data used in this paper are public data and can be obtained from NOAA through Amazon Web Services https://registry.opendata.aws/noaa-goes/. National Geographic images and Esri world imagery are from Esri ArcGIS. MODIS images and data are public and can be obtained from NASA as raw data or a geotiff. Earth Networks data were obtained by C.J.S. as part of the GOES GLM Calibration and Validation work during 2017 and 2018.
