# Supplementary material for: Observations of lightning in relation to transitions in volcanic activity during the 3 June 2018 Fuego Eruption
Source: Sci Rep. 2020 Oct 22;10:18015. doi: 10.1038/s41598-020-74576-x (PMC7582158; doi:10.1038/s41598-020-74576-x)
Supplement: Supplementary file 1 — Supplementary Information. [file 41598_2020_74576_MOESM1_ESM.docx]

**Supplementary Data**

**Observations of Lightning in Relation to Transitions in Volcanic Activity during the 3 June 2018 Fuego Eruption**

Christopher J. Schultz^1*^, Virginia Andrews^2^, Kimberly Genareau^2^, Aaron Naeger^3^

^1^NASA’s Short-term Prediction and Research Transition Center, Marshall Space Flight Center, Huntsville, Alabama, 35812

^2^Department of Geological Sciences, The University of Alabama, Box 870338, Tuscaloosa, AL 35487, USA

^3^Earth System Science Center, The University of Alabama in Huntsville, Huntsville, AL, USA

Corresponding author: [christopher.j.schultz@nasa.gov](mailto:christopher.j.schultz@nasa.gov)

**
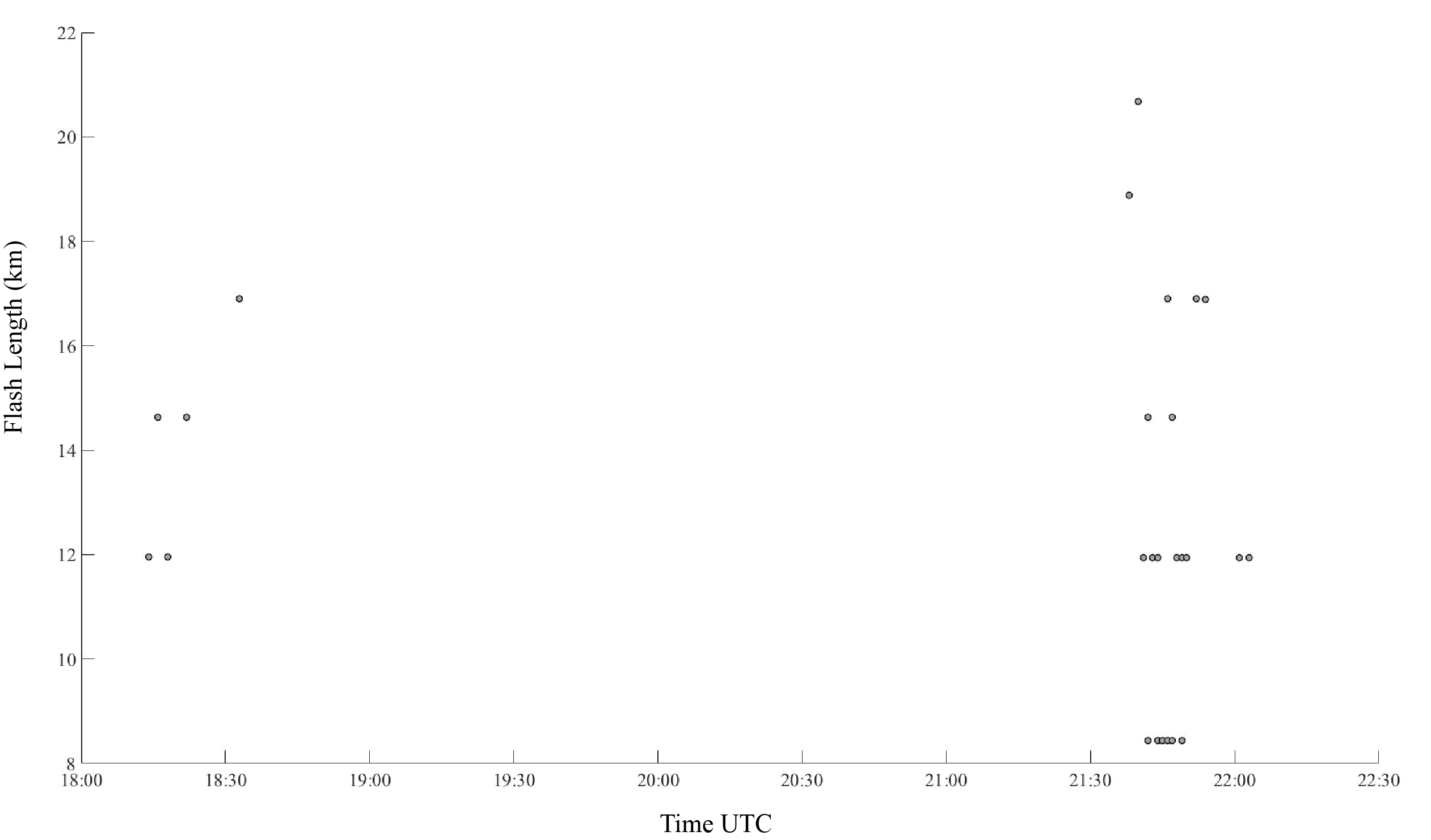
**

**Figure S1.** Geostationary Lightning Mapper (GLM) data during the 3 June 2018 eruption of Volcán de Fuego, Guatemala. Lightning flash length as a function of time to demonstrate the smaller flash lengths interpreted as lightning associated with the secondary maxima in lightning.


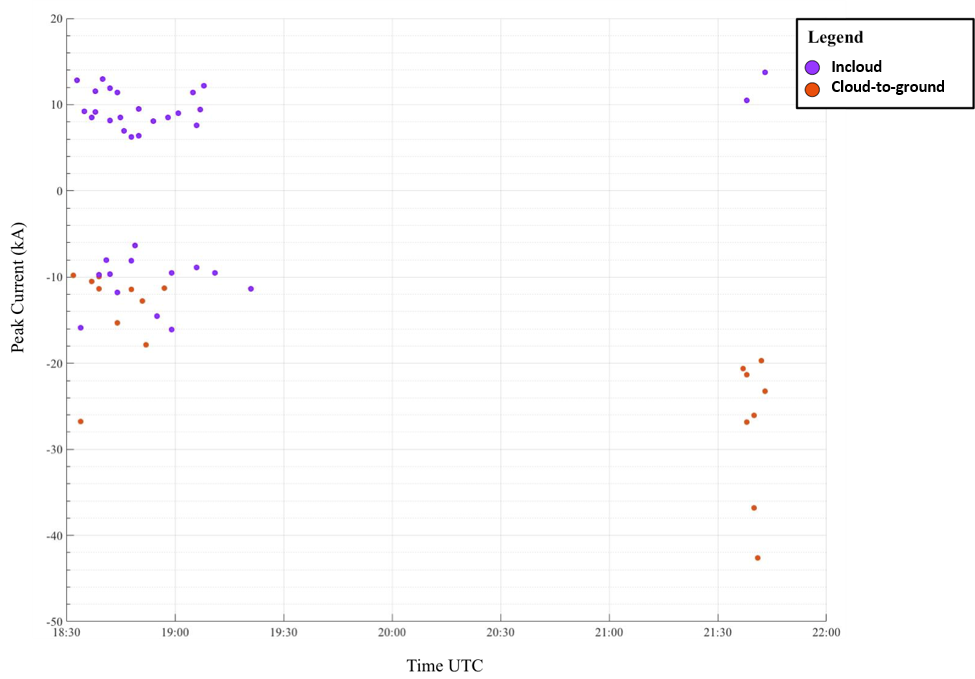


**Figure S2.** Earth Networks Total Lightning Network (ENTLN) data during the 3 June 2018 eruption of Volcán de Fuego, Guatemala, showing the peak current of both intra-cloud and cloud-to ground lightning discharges.

| Latitude | Longitude | Flash Area (km) | Time UTC | Flash Length (km) | Flash Energy (fJ) | Distance from Fuego Vent (km) |
| --- | --- | --- | --- | --- | --- | --- |
|  |  |  |  |  |  |  |
| 14.54320526 | -90.91100311 | 142.85786 | 1814000 | 11.9523 | 11 | 8.28 |
| 14.54983425 | -90.91503906 | 214.27983 | 1816400 | 14.6383 | 21 | 9.13 |
| 14.56776047 | -90.91296387 | 142.85786 | 1818200 | 11.9523 | 20 | 10.91 |
| 14.59312534 | -90.90883636 | 214.27983 | 1822200 | 14.6383 | 29 | 13.50 |
| 14.92485237 | -90.45609283 | 1498.9656 | 1831200 | 38.7165 | 1857 | 76.15 |
| 14.59567928 | -90.95252228 | 285.85345 | 1833000 | 16.9072 | 18 | 15.51 |
| 14.42387104 | -90.76902771 | 356.36559 | 2138000 | 18.8776 | 13 | 13.27 |
| 14.39133263 | -90.7555542 | 427.48429 | 2140400 | 20.6757 | 14 | 16.33 |
| 14.52069855 | -90.82458496 | 142.70622 | 2141200 | 11.946 | 8 | 7.90 |
| 14.51911736 | -90.81668091 | 71.28424 | 2142400 | 8.443 | 6 | 8.46 |
| 14.44631767 | -90.88090515 | 214.12819 | 2142400 | 14.6331 | 25 | 3.15 |
| 14.51952744 | -90.82348633 | 142.70622 | 2143200 | 11.946 | 9 | 7.91 |
| 14.44276333 | -90.80937958 | 71.28424 | 2144200 | 8.443 | 5 | 8.44 |
| 14.51947212 | -90.82456207 | 142.70622 | 2144400 | 11.946 | 8 | 7.82 |
| 14.51922607 | -90.81626892 | 71.28424 | 2145400 | 8.443 | 30 | 8.51 |
| 14.46459579 | -90.89370728 | 285.55017 | 2146200 | 16.8982 | 50 | 1.80 |
| 14.51999283 | -90.81616211 | 71.28424 | 2146400 | 8.443 | 8 | 8.57 |
| 14.43835354 | -90.81022644 | 213.97655 | 2147200 | 14.6279 | 13 | 8.58 |
| 14.51954651 | -90.81622314 | 71.28424 | 2147200 | 8.443 | 2 | 8.53 |
| 14.46605301 | -90.89073944 | 142.70622 | 2148200 | 11.946 | 21 | 1.45 |
| 14.51988792 | -90.81615448 | 71.28424 | 2149000 | 8.443 | 2 | 8.56 |
| 14.52007484 | -90.81664276 | 71.28424 | 2149400 | 8.443 | 9 | 8.53 |
| 14.49602032 | -90.89395142 | 142.70622 | 2149400 | 11.946 | 19 | 2.77 |
| 14.44244289 | -90.80547333 | 142.55458 | 2150000 | 11.9396 | 18 | 8.84 |
| 14.49525928 | -90.89331818 | 285.55017 | 2152000 | 16.8982 | 34 | 2.66 |
| 14.57121563 | -90.81598663 | 285.39853 | 2154400 | 16.8937 | 71 | 12.78 |
| 14.47427559 | -90.89032745 | 142.70622 | 2201400 | 11.946 | 32 | 1.05 |
| 14.51438332 | -90.89464569 | 142.70622 | 2203200 | 11.946 | 13 | 4.66 |

**Table S1.** Geostationary Lightning Mapper (GLM) data from the 3 June 2018 eruption of Volcán de Fuego, Guatemala. All data are from lightning occurring from 16:00 to 23:00 UTC within 30 km of the volcanic vent.

| Latitude | Longitude | Flash Area (km) | Time UTC | Flash Length (km) | Flash Energy (fJ) |
| --- | --- | --- | --- | --- | --- |
| 14.54320526 | -90.91100311 | 142.8579 | 1814000 | 11.9523 | 16.786 |
| 14.54983425 | -90.91503906 | 214.2798 | 1816400 | 14.6383 | 32.045 |
| 14.56776047 | -90.91296387 | 142.8579 | 1818200 | 11.9523 | 30.519 |
| 14.59312534 | -90.90883636 | 214.2798 | 1822200 | 14.6383 | 44.253 |
| 14.92485237 | -90.45609283 | 1498.9656 | 1831200 | 38.7165 | 1857.105 |
| 14.59567928 | -90.95252228 | 285.8534 | 1833000 | 16.9072 | 27.467 |
| 13.90178204 | -89.8263855 | 282.5174 | 1956000 | 16.8083 | 70.195 |
| 13.91663074 | -89.83316803 | 494.3571 | 1957000 | 22.2341 | 463.895 |
| 13.90043354 | -89.82250214 | 211.8536 | 1958200 | 14.5552 | 100.714 |
| 13.90993214 | -89.75299072 | 1552.7974 | 2000200 | 39.4056 | 227.37 |
| 13.93411541 | -89.80331421 | 1552.3425 | 2002000 | 39.3998 | 355.551 |
| 13.9230175 | -89.8351059 | 776.8606 | 2003200 | 27.8722 | 292.986 |
| 13.91956711 | -89.83732605 | 847.0694 | 2005400 | 29.1045 | 227.37 |
| 13.96648693 | -89.87954712 | 706.6517 | 2007000 | 26.5829 | 387.596 |
| 13.94143963 | -89.82707977 | 282.3657 | 2009400 | 16.8037 | 201.428 |
| 14.99261951 | -91.66363525 | 72.0424 | 2028000 | 8.4878 | 16.786 |
| 14.99249649 | -91.68183899 | 144.071 | 2032200 | 12.003 | 35.097 |
| 14.0766592 | -90.49007416 | 284.0338 | 2103400 | 16.8533 | 12.208 |
| 14.07795238 | -90.49192047 | 284.0338 | 2104200 | 16.8533 | 41.201 |
| 14.05448246 | -90.4601059 | 70.981 | 2105000 | 8.425 | 4.578 |
| 14.114254 | -90.46498108 | 141.948 | 2109200 | 11.9142 | 7.63 |
| 14.05430889 | -90.457901 | 70.981 | 2110000 | 8.425 | 4.578 |
| 14.07993031 | -90.49559021 | 1207.3637 | 2110200 | 34.7471 | 265.519 |
| 14.13247967 | -90.47788239 | 142.0997 | 2110400 | 11.9206 | 18.312 |
| 14.08760452 | -90.48927307 | 355.1525 | 2112000 | 18.8455 | 57.987 |
| 14.13271904 | -90.49503326 | 142.0997 | 2112200 | 11.9206 | 19.838 |
| 14.05391884 | -90.4741745 | 212.9151 | 2113400 | 14.5916 | 70.195 |
| 14.07180119 | -90.53878021 | 142.0997 | 2114000 | 11.9206 | 7.63 |
| 14.05768394 | -90.48841095 | 567.902 | 2115000 | 23.8307 | 109.87 |
| 14.14323997 | -90.54169464 | 1208.4252 | 2116000 | 34.7624 | 379.967 |
| 14.07304955 | -90.48923492 | 284.0338 | 2117000 | 16.8533 | 24.416 |
| 14.08715153 | -90.49344635 | 284.0338 | 2117200 | 16.8533 | 27.467 |
| 14.19663429 | -90.39299774 | 141.948 | 2117400 | 11.9142 | 33.571 |
| 14.07511902 | -90.53141785 | 284.0338 | 2117400 | 16.8533 | 39.675 |
| 14.06870556 | -90.49224091 | 212.9151 | 2118000 | 14.5916 | 12.208 |
| 14.0678339 | -90.52186584 | 284.0338 | 2118200 | 16.8533 | 24.416 |
| 14.09436035 | -90.52433014 | 923.4955 | 2119000 | 30.3891 | 384.544 |
| 14.08223248 | -90.52381134 | 284.0338 | 2119400 | 16.8533 | 65.617 |
| 14.07728958 | -90.51134491 | 284.0338 | 2120000 | 16.8533 | 24.416 |
| 14.0844593 | -90.55171967 | 1279.2406 | 2120400 | 35.7665 | 665.323 |
| 14.06447697 | -90.4826355 | 212.9151 | 2120400 | 14.5916 | 15.26 |
| 14.0868206 | -90.51100159 | 284.0338 | 2121200 | 16.8533 | 105.292 |
| 14.0942812 | -90.46432495 | 213.0667 | 2121400 | 14.5968 | 62.565 |
| 14.0931797 | -90.51964569 | 284.0338 | 2122000 | 16.8533 | 36.623 |
| 14.09300327 | -90.49555969 | 284.0338 | 2122400 | 16.8533 | 36.623 |
| 14.10839367 | -90.50125122 | 284.0338 | 2123000 | 16.8533 | 120.552 |
| 14.10987473 | -90.46404266 | 141.948 | 2123400 | 11.9142 | 15.26 |
| 14.12837696 | -90.49074554 | 213.0667 | 2124200 | 14.5968 | 44.253 |
| 14.12456989 | -90.51818848 | 213.0667 | 2124400 | 14.5968 | 16.786 |
| 14.13141632 | -90.49944305 | 142.0997 | 2125000 | 11.9206 | 44.253 |
| 14.09731293 | -90.5417099 | 142.0997 | 2125200 | 11.9206 | 9.156 |
| 14.13338375 | -90.49488068 | 213.0667 | 2126000 | 14.5968 | 96.136 |
| 14.13228321 | -90.51073456 | 142.0997 | 2126200 | 11.9206 | 44.253 |
| 14.11733627 | -90.5085144 | 284.0338 | 2126400 | 16.8533 | 48.831 |
| 14.13698387 | -90.49559784 | 284.1854 | 2127000 | 16.8578 | 117.5 |
| 14.11702442 | -90.49269867 | 284.0338 | 2127400 | 16.8533 | 56.461 |
| 14.1632967 | -90.53404999 | 1563.4121 | 2128200 | 39.54 | 1008.666 |
| 14.1639986 | -90.53871155 | 1635.5923 | 2129200 | 40.4425 | 630.226 |
| 14.14532471 | -90.51564789 | 781.4097 | 2130000 | 27.9537 | 68.669 |
| 14.13704586 | -90.52207947 | 426.4228 | 2130400 | 20.65 | 331.135 |
| 14.1316433 | -90.47531891 | 142.0997 | 2131200 | 11.9206 | 59.513 |
| 14.13921928 | -90.50930786 | 568.3569 | 2132000 | 23.8402 | 280.778 |
| 14.14281273 | -90.52485657 | 355.3041 | 2132200 | 18.8495 | 44.253 |
| 14.12108517 | -90.46906281 | 213.0667 | 2132400 | 14.5968 | 41.201 |
| 14.14309597 | -90.48683929 | 355.1525 | 2133000 | 18.8455 | 183.116 |
| 14.15364075 | -90.38829041 | 141.948 | 2133200 | 11.9142 | 6.104 |
| 14.13235283 | -90.48334503 | 426.1195 | 2134000 | 20.6427 | 373.863 |
| 14.13345909 | -90.46896362 | 213.0667 | 2134400 | 14.5968 | 80.876 |
| 14.1439867 | -90.47192383 | 213.0667 | 2135200 | 14.5968 | 111.396 |
| 14.12043381 | -90.46520233 | 496.935 | 2135400 | 22.292 | 134.285 |
| 14.13700962 | -90.46772766 | 213.0667 | 2136400 | 14.5968 | 99.188 |
| 14.1540184 | -90.43392181 | 284.0338 | 2137200 | 16.8533 | 19.838 |
| 14.13214684 | -90.46573639 | 142.0997 | 2137400 | 11.9206 | 140.389 |
| 14.42387104 | -90.76902771 | 356.3656 | 2138000 | 18.8776 | 21.364 |
| 14.13161278 | -90.46398163 | 70.981 | 2138400 | 8.425 | 48.831 |
| 14.14091873 | -90.47816467 | 355.1525 | 2139200 | 18.8455 | 280.778 |
| 14.15057087 | -90.46710205 | 142.0997 | 2140000 | 11.9206 | 28.993 |
| 14.39133263 | -90.7555542 | 427.4843 | 2140400 | 20.6757 | 19.838 |
| 14.16487694 | -90.47232819 | 639.4756 | 2141000 | 25.2879 | 346.395 |
| 14.52069855 | -90.82458496 | 142.7062 | 2141200 | 11.946 | 12.208 |
| 14.20705986 | -90.40908051 | 142.0997 | 2141400 | 11.9206 | 12.208 |
| 14.18126869 | -90.46854401 | 142.0997 | 2142000 | 11.9206 | 77.824 |
| 14.95309448 | -91.88592529 | 432.7917 | 2142400 | 20.8036 | 56.461 |
| 14.51911736 | -90.81668091 | 71.2842 | 2142400 | 8.443 | 9.156 |
| 14.44631767 | -90.88090515 | 214.1282 | 2142400 | 14.6331 | 38.149 |
| 14.19154835 | -90.41581726 | 213.0667 | 2143200 | 14.5968 | 79.35 |
| 14.18575764 | -90.46907806 | 142.0997 | 2143200 | 11.9206 | 80.876 |
| 14.51952744 | -90.82348633 | 142.7062 | 2143200 | 11.946 | 13.734 |
| 14.44276333 | -90.80937958 | 71.2842 | 2144200 | 8.443 | 7.63 |
| 14.19050217 | -90.470047 | 142.0997 | 2144200 | 11.9206 | 30.519 |
| 14.51947212 | -90.82456207 | 142.7062 | 2144400 | 11.946 | 12.208 |
| 14.20703411 | -90.43754578 | 142.0997 | 2145000 | 11.9206 | 21.364 |
| 14.20196056 | -90.47055054 | 142.0997 | 2145400 | 11.9206 | 21.364 |
| 14.51922607 | -90.81626892 | 71.2842 | 2145400 | 8.443 | 45.779 |
| 14.20858097 | -90.46522522 | 142.0997 | 2146000 | 11.9206 | 76.298 |
| 14.46459579 | -90.89370728 | 285.5502 | 2146200 | 16.8982 | 76.298 |
| 14.51999283 | -90.81616211 | 71.2842 | 2146400 | 8.443 | 12.208 |
| 14.98927784 | -91.89587402 | 216.4028 | 2147000 | 14.7106 | 73.247 |
| 14.20528603 | -90.46958923 | 213.0667 | 2147200 | 14.5968 | 80.876 |
| 14.43835354 | -90.81022644 | 213.9766 | 2147200 | 14.6279 | 19.838 |
| 14.51954651 | -90.81622314 | 71.2842 | 2147200 | 8.443 | 3.052 |
| 14.46605301 | -90.89073944 | 142.7062 | 2148200 | 11.946 | 32.045 |
| 14.20711994 | -90.46630859 | 213.0667 | 2148400 | 14.5968 | 79.35 |
| 14.98378468 | -91.90031433 | 144.2226 | 2149000 | 12.0093 | 33.571 |
| 14.51988792 | -90.81615448 | 71.2842 | 2149000 | 8.443 | 3.052 |
| 14.52007484 | -90.81664276 | 71.2842 | 2149400 | 8.443 | 13.734 |
| 14.49602032 | -90.89395142 | 142.7062 | 2149400 | 11.946 | 28.993 |
| 14.44244289 | -90.80547333 | 142.5546 | 2150000 | 11.9396 | 27.467 |
| 14.20919418 | -90.47089386 | 70.981 | 2150000 | 8.425 | 57.987 |
| 14.20745564 | -90.47130585 | 70.981 | 2151200 | 8.425 | 30.519 |
| 14.99668694 | -91.89997101 | 72.1941 | 2151400 | 8.4967 | 16.786 |
| 14.49525928 | -90.89331818 | 285.5502 | 2152000 | 16.8982 | 51.883 |
| 14.9961338 | -91.90106964 | 72.1941 | 2153400 | 8.4967 | 10.682 |
| 14.21056366 | -90.45845795 | 213.0667 | 2154200 | 14.5968 | 128.181 |
| 14.57121563 | -90.81598663 | 285.3985 | 2154400 | 16.8937 | 108.344 |
| 14.96581554 | -91.90466309 | 504.9718 | 2155200 | 22.4716 | 326.558 |
| 14.99285126 | -91.89601135 | 216.2511 | 2157400 | 14.7055 | 53.409 |
| 14.95481396 | -91.88326263 | 504.8202 | 2201200 | 22.4682 | 421.168 |
| 14.47427559 | -90.89032745 | 142.7062 | 2201400 | 11.946 | 48.831 |
| 14.51438332 | -90.89464569 | 142.7062 | 2203200 | 11.946 | 19.838 |
| 14.97099876 | -91.89019012 | 721.2091 | 2205000 | 26.8553 | 152.597 |
| 14.99281406 | -91.90255737 | 216.4028 | 2206000 | 14.7106 | 112.922 |
| 14.99587917 | -91.89996338 | 72.1941 | 2207200 | 8.4967 | 7.63 |
| 14.98513126 | -91.89458466 | 432.64 | 2209000 | 20.8 | 251.785 |
| 14.55487633 | -91.31102753 | 286.6116 | 2212400 | 16.9296 | 16.786 |
| 14.99648857 | -91.91533661 | 288.7346 | 2212400 | 16.9922 | 192.272 |
| 14.98858356 | -91.90660095 | 288.5829 | 2214000 | 16.9877 | 50.357 |
| 14.60221863 | -91.30056 | 71.5875 | 2217200 | 8.4609 | 7.63 |
| 14.99607086 | -91.91796112 | 144.3742 | 2218000 | 12.0156 | 12.208 |
| 14.5632658 | -91.33694458 | 286.6116 | 2219000 | 16.9296 | 13.734 |
| 14.59531212 | -91.33783722 | 214.8864 | 2221200 | 14.659 | 16.786 |
| 14.61202908 | -91.3560257 | 1146.5565 | 2223000 | 33.8608 | 642.433 |
| 14.6167593 | -91.37078857 | 1792.0838 | 2225200 | 42.333 | 634.804 |
| 14.57761669 | -91.32396698 | 358.1853 | 2227200 | 18.9258 | 51.883 |
| 14.56763744 | -91.3259201 | 358.1853 | 2230000 | 18.9258 | 114.448 |
| 14.4496336 | -91.44454956 | 71.5875 | 2251400 | 8.4609 | 4.578 |
| 14.46253777 | -91.42836761 | 214.8864 | 2252200 | 14.659 | 7.63 |
| 14.46674919 | -91.36952209 | 429.7589 | 2253000 | 20.7306 | 18.312 |
| 14.49817562 | -91.42642975 | 215.038 | 2254000 | 14.6642 | 10.682 |
| 14.46910191 | -91.33118439 | 1073.6181 | 2254400 | 32.7661 | 77.824 |
| 14.49421406 | -91.42601776 | 286.6116 | 2255400 | 16.9296 | 12.208 |
| 14.45267487 | -91.44821167 | 215.038 | 2256400 | 14.6642 | 39.675 |
| 14.46307659 | -91.43807983 | 358.3369 | 2257000 | 18.9298 | 38.149 |
| 14.46088409 | -91.44498444 | 143.3128 | 2257200 | 11.9713 | 21.364 |
| 14.53384876 | -91.43022919 | 215.038 | 2257400 | 14.6642 | 18.312 |
| 14.45305061 | -91.43212128 | 214.8864 | 2258000 | 14.659 | 30.519 |
| 14.50143337 | -91.42075348 | 573.2094 | 2258400 | 23.9418 | 67.143 |
| 14.46632671 | -91.39807129 | 429.6072 | 2259000 | 20.727 | 25.941 |
| 14.46204853 | -91.42020416 | 429.7589 | 2259200 | 20.7306 | 61.039 |
| 14.4628334 | -91.4128952 | 286.6116 | 2259400 | 16.9296 | 19.838 |
| 14.84027576 | -91.88250732 | 72.0424 | 2300000 | 8.4878 | 6.104 |
| 14.52359962 | -91.41235352 | 214.8864 | 2300200 | 14.659 | 39.675 |
| 14.4735527 | -91.42068481 | 358.3369 | 2301000 | 18.9298 | 39.675 |
| 14.91725254 | -91.88988495 | 72.0424 | 2301200 | 8.4878 | 4.578 |
| 14.46892262 | -91.37966156 | 1002.4994 | 2301200 | 31.6623 | 70.195 |
| 14.4881382 | -91.36798859 | 143.3128 | 2301400 | 11.9713 | 15.26 |
| 14.5265789 | -91.42492676 | 143.3128 | 2302000 | 11.9713 | 9.156 |
| 14.91353512 | -91.91013336 | 937.7496 | 2302200 | 30.6227 | 198.376 |
| 14.48943996 | -91.38316345 | 286.6116 | 2302200 | 16.9296 | 15.26 |
| 14.44939327 | -91.44346619 | 71.5875 | 2302400 | 8.4609 | 6.104 |
| 14.50662804 | -91.46094513 | 1576.7564 | 2303400 | 39.7084 | 698.894 |
| 14.44892502 | -91.43797302 | 143.3128 | 2304000 | 11.9713 | 19.838 |
| 14.85587978 | -91.89709473 | 1658.0349 | 2305000 | 40.719 | 622.596 |
| 14.4669981 | -91.42773438 | 143.3128 | 2305200 | 11.9713 | 6.104 |
| 14.49525833 | -91.44406128 | 501.6358 | 2306000 | 22.3972 | 76.298 |
| 14.50131416 | -91.42593384 | 286.6116 | 2306400 | 16.9296 | 25.941 |
| 14.4632206 | -91.46062469 | 573.2094 | 2307000 | 23.9418 | 61.039 |
| 14.93814182 | -91.91299438 | 721.3607 | 2307200 | 26.8582 | 538.667 |
| 14.52560616 | -91.37139893 | 71.5875 | 2307200 | 8.4609 | 6.104 |
| 14.49902821 | -91.43539429 | 501.6358 | 2308200 | 22.3972 | 137.337 |
| 14.90431023 | -91.89208221 | 216.2511 | 2309400 | 14.7055 | 48.831 |
| 14.50269604 | -91.43357086 | 286.6116 | 2309400 | 16.9296 | 51.883 |
| 14.53701019 | -91.43586731 | 358.3369 | 2310400 | 18.9298 | 16.786 |
| 14.91493225 | -91.89363098 | 216.2511 | 2311000 | 14.7055 | 85.454 |
| 14.51490498 | -91.40216827 | 286.6116 | 2311200 | 16.9296 | 93.084 |
| 14.52610588 | -91.45054626 | 71.7392 | 2312200 | 8.4699 | 38.149 |
| 14.91723251 | -91.89334106 | 144.2226 | 2312400 | 12.0093 | 41.201 |
| 14.91693115 | -91.88924408 | 72.0424 | 2314200 | 8.4878 | 28.993 |
| 14.4489212 | -91.44313812 | 71.5875 | 2314200 | 8.4609 | 16.786 |
| 14.51949024 | -91.4358902 | 358.4885 | 2315000 | 18.9338 | 120.552 |
| 14.91668606 | -91.88918304 | 72.0424 | 2315200 | 8.4878 | 38.149 |
| 14.46717453 | -91.44515991 | 143.3128 | 2315200 | 11.9713 | 7.63 |
| 14.54410934 | -91.47570801 | 1291.3718 | 2316400 | 35.9357 | 383.018 |
| 14.45666313 | -91.43947601 | 214.8864 | 2317200 | 14.659 | 16.786 |
| 14.89179611 | -91.93080139 | 937.7496 | 2317400 | 30.6227 | 213.636 |
| 14.53959465 | -91.48973846 | 1291.3718 | 2319200 | 35.9357 | 634.804 |
| 14.53469181 | -91.45413208 | 358.4885 | 2321000 | 18.9338 | 141.915 |
| 14.91139126 | -91.88943481 | 144.2226 | 2321000 | 12.0093 | 27.467 |
| 14.90684223 | -91.88198853 | 432.4884 | 2322400 | 20.7964 | 36.623 |
| 14.49034119 | -91.40181732 | 501.3325 | 2323200 | 22.3905 | 102.24 |
| 14.92387009 | -91.8924408 | 432.64 | 2327000 | 20.8 | 114.448 |
| 14.91896248 | -91.90041351 | 216.4028 | 2330400 | 14.7106 | 68.669 |
| 14.93953705 | -91.89661407 | 216.4028 | 2336400 | 14.7106 | 61.039 |
| 14.51071548 | -91.48192596 | 860.262 | 2338000 | 29.3302 | 508.148 |
| 14.45013618 | -91.52254486 | 71.7392 | 2339400 | 8.4699 | 3.052 |
| 14.51362991 | -91.52880859 | 1076.0444 | 2341200 | 32.8031 | 473.051 |
| 14.4895153 | -91.53726959 | 215.1897 | 2343000 | 14.6693 | 9.156 |
| 14.48630047 | -91.53822327 | 430.2138 | 2343200 | 20.7416 | 39.675 |
| 14.483881 | -91.52581024 | 143.4644 | 2344400 | 11.9777 | 7.63 |
| 14.46872997 | -91.53196716 | 286.7633 | 2345000 | 16.9341 | 21.364 |
| 14.4978447 | -91.49945068 | 1075.2862 | 2345200 | 32.7916 | 514.252 |
| 14.47047043 | -91.52410889 | 143.4644 | 2345400 | 11.9777 | 6.104 |
| 14.48490238 | -91.5355072 | 501.939 | 2346200 | 22.404 | 103.766 |
| 14.49644375 | -91.58100128 | 645.8445 | 2346400 | 25.4135 | 80.876 |
| 14.48125839 | -91.54647827 | 286.9149 | 2347000 | 16.9386 | 68.669 |
| 14.49085903 | -91.55039215 | 860.7169 | 2347400 | 29.338 | 186.168 |
| 14.48129272 | -91.54508209 | 860.5653 | 2348200 | 29.3354 | 331.135 |
| 14.5000658 | -91.55011749 | 502.2423 | 2348400 | 22.4108 | 132.759 |
| 14.48453045 | -91.55652618 | 1004.3191 | 2349000 | 31.691 | 292.986 |
| 14.48239326 | -91.54393768 | 358.6402 | 2349200 | 18.9378 | 62.565 |
| 14.48117161 | -91.56891632 | 286.9149 | 2349400 | 16.9386 | 15.26 |
| 14.46910954 | -91.55289459 | 573.6643 | 2350000 | 23.9513 | 61.039 |
| 14.50260162 | -91.57510376 | 286.9149 | 2350000 | 16.9386 | 18.312 |
| 14.45536041 | -91.54138184 | 716.9632 | 2350200 | 26.7762 | 338.765 |
| 14.47146702 | -91.7076416 | 430.8203 | 2350400 | 20.7562 | 56.461 |
| 14.48343754 | -91.55874634 | 286.9149 | 2351000 | 16.9386 | 10.682 |
| 14.47270393 | -91.52799988 | 215.1897 | 2351200 | 14.6693 | 32.045 |
| 14.47462845 | -91.56700897 | 286.9149 | 2351200 | 16.9386 | 50.357 |
| 14.49240398 | -91.5662384 | 502.0907 | 2351400 | 22.4074 | 155.649 |
| 14.48203087 | -91.58382416 | 861.0202 | 2352000 | 29.3431 | 265.519 |
| 14.51032829 | -91.57711029 | 502.394 | 2352200 | 22.4141 | 292.986 |
| 14.47940063 | -91.54697418 | 932.4422 | 2352400 | 30.5359 | 234.999 |
| 14.49550343 | -91.55661011 | 717.1148 | 2353200 | 26.779 | 166.331 |
| 14.518857 | -91.57408905 | 573.9676 | 2353400 | 23.9576 | 22.89 |
| 14.49075508 | -91.56092072 | 430.5171 | 2354000 | 20.7489 | 123.604 |
| 14.45551395 | -91.52372742 | 358.4885 | 2354200 | 18.9338 | 53.409 |
| 14.55288315 | -91.56484222 | 1363.7036 | 2354400 | 36.9284 | 1037.66 |
| 14.46396732 | -91.54530334 | 358.6402 | 2355000 | 18.9378 | 64.091 |
| 14.51770401 | -91.5591507 | 430.5171 | 2355400 | 20.7489 | 83.928 |
| 14.54311562 | -91.58720398 | 1579.031 | 2356200 | 39.737 | 509.674 |
| 14.52022171 | -91.57294464 | 430.5171 | 2356400 | 20.7489 | 47.305 |
| 14.53854084 | -91.60741425 | 933.352 | 2357200 | 30.5508 | 361.655 |
| 14.51589108 | -91.55384827 | 573.8159 | 2358000 | 23.9545 | 251.785 |
| 14.52513123 | -91.60199738 | 932.8971 | 2358200 | 30.5434 | 259.415 |
| 14.58099079 | -91.56414032 | 1794.8133 | 2358400 | 42.3652 | 842.335 |
| 14.51839542 | -91.59812164 | 358.7918 | 2359000 | 18.9418 | 36.623 |
| 14.45457935 | -91.53361511 | 573.5127 | 2359200 | 23.9481 | 97.662 |
| 14.52135563 | -91.61270142 | 358.7918 | 2359200 | 18.9418 | 57.987 |
| 14.5321064 | -91.60295105 | 1076.6509 | 2359400 | 32.8124 | 202.954 |

**Table S2.** Geostationary Lightning Mapper (GLM) data from the 3 June 2018 eruption of Volcán de Fuego, Guatemala. All data are from lightning occurring from 16:00 to 23:00 within 120 km of the volcanic vent.
